# Infrared Spectrometry as a High-Throughput Phenotyping Technology to Predict Complex Traits in Livestock Systems

**DOI:** 10.3389/fgene.2020.00923

**Published:** 2020-08-20

**Authors:** Tiago Bresolin, João R. R. Dórea

**Affiliations:** Department of Animal and Dairy Sciences, University of Wisconsin-Madison, Madison, WI, United States

**Keywords:** beef cattle, dairy cattle, near-infrared, novel phenotypes, mid-infrared, spectral information

## Abstract

High-throughput phenotyping technologies are growing in importance in livestock systems due to their ability to generate real-time, non-invasive, and accurate animal-level information. Collecting such individual-level information can generate novel traits and potentially improve animal selection and management decisions in livestock operations. One of the most relevant tools used in the dairy and beef industry to predict complex traits is infrared spectrometry, which is based on the analysis of the interaction between electromagnetic radiation and matter. The infrared electromagnetic radiation spans an enormous range of wavelengths and frequencies known as the electromagnetic spectrum. The spectrum is divided into different regions, with near- and mid-infrared regions being the main spectral regions used in livestock applications. The advantage of using infrared spectrometry includes speed, non-destructive measurement, and great potential for on-line analysis. This paper aims to review the use of mid- and near-infrared spectrometry techniques as tools to predict complex dairy and beef phenotypes, such as milk composition, feed efficiency, methane emission, fertility, energy balance, health status, and meat quality traits. Although several research studies have used these technologies to predict a wide range of phenotypes, most of them are based on Partial Least Squares (PLS) and did not considered other machine learning (ML) techniques to improve prediction quality. Therefore, we will discuss the role of analytical methods employed on spectral data to improve the predictive ability for complex traits in livestock operations. Furthermore, we will discuss different approaches to reduce data dimensionality and the impact of validation strategies on predictive quality.

## Introduction

For many years dairy and beef cattle breeding have focused on improving the production and profitability of animals through genetics, nutrition, and management, often at the expense of other relevant traits. To remain competitive and meet the world population increase and global climate changes, farmers need to balance production, profitability, and sustainability. There is an extensive list of key phenotypes that must be measured to achieve the emerging breeding goals for the advance of genomic selection (Boichard and Brochard, 2012) and management decisions in the context of precision agriculture. However, recording such phenotypes in large-scale or across different herds and countries is a challenge (Gengler et al., 2016). High-throughput phenotyping technologies have grown in importance in livestock systems because of their ability to generate real-time and accurate animal-level information. Several technologies (e.g., sensors, infrared spectrometry, and image analysis, among others) have been used to generate novel complex traits in dairy and beef cattle, with infrared spectrometry being one of the most relevant tools used in livestock to date (De Marchi et al., 2014; Dixit et al., 2017; Bell and Tzimiropoulos, 2018). Infrared spectrometry is based on the interaction between electromagnetic radiation (infrared light) and matter. The modern Fourier transform infrared spectrometers spans an enormous range of infrared spectrum, which is divided into three main regions: NIR, near-infrared (800–2,500 nm or 4,000–12,500 cm^–1^); MIR, mid-infrared (2,500–25,000 nm or 400–4,000 cm^–1^); and FAR, far-infrared (25,000–1,000,000 nm or 10–400 cm^–1^). NIR and MIR are the main regions used in livestock applications (Griffiths and de Hasenth, 2007). This technology is fast, non-invasive, non-destructive, and has great potential for on-line measurement (De Marchi et al., 2014; Dixit et al., 2017).

Infrared spectrometry, mainly MIR, has been widely used worldwide to predict the concentration of protein, casein, fat, lactose, and urea of milk through regular recording schemes (De Marchi et al., 2014). When cows are milked 2–3 times daily, this biological sample can be more deeply interrogated to generate novel complex traits, which are usually expensive and difficult to be measure on a large scale (e.g., individual milk fatty acids, proteins, feed intake, methane emission, fertility, energy balance, health status, and others). The majority of milk constituents synthesized in the mammary gland are based on the by-products from the digestion of the nutrients ingested in a given day (McParland and Berry, 2016). Therefore, changes in milk composition profile on that day or in the following days can be used as a biomarker for complex phenotypes related to metabolism. Within the beef industry, NIR technology has been shown to be a valuable and cost-effective technology to assess several meat quality attributes (e.g., tenderness, fat content, color, among others) at the same time without any or minimal sample preparation and pretreatment (Prieto et al., 2009a; Dixit et al., 2017; Chapman et al., 2019). Therefore, the NIR technique can be applied directly to the samples, which is an advantage compared to reference methods, and it is also important for the slaughterhouses that can reduce losses with carcass sample assessment and sample preparation. Both technologies (NIR and MIR) have great potential to assess different milk or meat attributes using in-line systems, which could lend deep insights and added efficiencies for both the dairy and beef industries.

Several authors have successfully used infrared spectrometry to predict a range number of traits as reported in previous reviews papers (Prevolnik et al., 2004; Prieto et al., 2009a, 2017; De Marchi et al., 2014; McParland and Berry, 2016; Dixit et al., 2017; Chapman et al., 2019). Although the aforementioned reviews discussed the use of MIR and NIR spectrometry as a tool to predict milk and meat traits, very little attention has been given to analytical methods and validation strategies employed in analyzing such spectral data. Thus, complementary to the previous reviews, the objectives of this review are: to provide a recent update on the use of MIR and NIR techniques as tools to predict several novel complex traits in livestock system, with an emphasis in dairy and beef cattle; and review and discuss the analytical methods employed on spectral data to improve predictive ability, the different approaches used to reduce data dimensionality, and the impact of validation strategies on the prediction quality.

## Methodology

For this review, research articles published in peer-reviewed journals were retrieved from Web of Science using the keywords or the random combination of keywords presented in Table 1. Initially, a total of 348 papers published until May 2020 was found. The papers using NIR or MIR were selected based on the phenotype of interest (e.g., milk composition, feed intake, energy balance, methane emission, fertility, health status, and meat quality traits), and from the 348 studies, only 113 were included in this review. Studies using pre-calibrated or pre-trained models, provided by a company or third party, were not considered in this review. The coefficient of determination (*R*^2^) was used as an indicator of prediction quality for continuous variables. Some authors used the correlation coefficient as one of the metrics to report the model prediction quality. As such, the correlation coefficients were converted to *R*^2^ in order to have a single statistical metric for model evaluation. Although we have adopted *R*^2^ as metric to evaluate prediction quality in this review, due to the number of phenotypes and studies, we also recognize that other important metrics, such as Root Mean Squared Error and Mean Absolute Error, must be considered to better evaluate prediction quality, once *R*^2^ could be inflated by one sample or it could be very sensitive to the range of the variable of interest. For discrete distributed traits, the metric for prediction quality, reviewed in the published papers, was the overall accuracy. Thus, only the *R*^2^ or accuracy reported by the author in the validation set (internal or external) were considered in this review paper. The validation strategy employed by each author (i.e., data-splitting, leave-one-out cross, or k-folds cross-validation) was reviewed and it is presented in the tables, along with the information of prediction quality for each respective phenotype. However, some papers reviewed here did not completely describe the validation strategy adopted; therefore, it was not reported in the tables of this review. More information related to the compiled validation strategies is presented in the section “Validation Strategies.”

**TABLE 1:** Keywords used to retrieve published papers from Web of Science*.

Cattle	Mid or Near-infrared
Dairy	Milk compounds, milk fatty acids, protein, minerals, metabolic status, energy balance, feed efficiency, feed intake, energy intake, methane emission, reproduction, fertility, lameness, blood metabolites
Beef	Meat quality, feed efficiency, feed intake, energy intake, methane emission, metabolic status, energy balance, reproduction, fertility

*Random combination of mid or near-infrared with the target phenotypes for beef and dairy cattle.

## Complex Traits Predicted by Infrared Spectrometry Data

**FIGURE 1:**
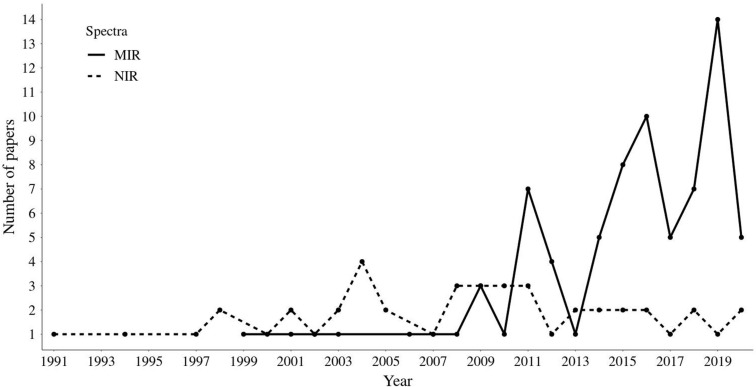
Published papers retrieved from Web of Science based on the combination of keywords presented in Table 1. Scientific papers published up to May 2020.

Over the past several years, many studies have investigated the effectiveness of NIR and MIR to predict novel complex phenotypes in dairy and beef cattle, as shown in Figure 1. Since De Marchi et al. (2014) wrote a review paper on milk MIR spectrometry, there has been an exponential increase in the number of studies using milk MIR spectral data to predict a range of complex traits. Overall, these studies include the direct quantification of compounds present in milk (e.g., milk fatty acids and protein profile) as well as the prediction of traits linked to milk spectra (e.g., health status, feed intake, methane emission, fertility, and energy balance). The use of NIR spectrometry to assess meat chemical composition and quality traits was reviewed for different species including beef, chicken, and pork by many authors (Prevolnik et al., 2004; Prieto et al., 2009a, 2017; Dixit et al., 2017; Chapman et al., 2019). The authors have stated that NIR is capable of measuring meat chemical composition and quality associated traits in different species, including beef cattle. However, the interest in using NIR technology as an alternative to predict novel traits in beef cattle since the first review paper (Prevolnik et al., 2004) has been lower than for using MIR spectrometry, based on the amount of paper using each method compiled for this current review (Figure 1). The number of studies developed in the last years using MIR and NIR techniques highlights the growing interest by the scientific community and livestock industry in this topic. Indeed, several novel phenotypes have been recently generated using both MIR and NIR techniques in dairy and beef cattle and they will be covered throughout this review section.

### Milk Composition

Beyond the nutritional meaningful for human, milk composition (e.g., protein, fat, lactose, and minerals) has direct implications on the sensory and technological properties of milk products as well as on the economic value of the milk and milk products (Soyeurt et al., 2009; Bastin et al., 2011; Bonfatti et al., 2011; Gengler et al., 2016; Fleming et al., 2017). Thus, over the last few years, efforts have been made by scientists and the dairy industry to quantify milk composition using modern high-throughput phenotyping techniques such as MIR spectrometry. Indeed, milk recording schemes worldwide have used MIR technique to measure total fat, protein, casein, lactose, and urea contents, which is quick and inexpensive when compared with gold standard methods (De Marchi et al., 2014). Given the promising and availability of milk spectra per cow per milking, several studies have reported that the major milk fatty acids (FA) can also be predicted using spectra data (Tables 2, 3).

**TABLE 2:** Number of samples (N) and coefficient of determination in the validation set for the milk fatty acids predicted from mid-infrared spectrometry using partial least square methodology in dairy cattle.

References	N	Breed	Validation*	C4:0	C6:0	C8:0	C10:0	C12:0	C14:0	C16:0	C16:1
Soyeurt et al. (2006)	49	Mul	CV	0.51	0.52	0.59	0.64	0.74	0.82	0.82	–
Soyeurt et al. (2008)	78	Mul	LOOCV	–	–	–	–	–	0.90	0.84	–
Rutten et al. (2009)	3,622	–	R-Tr/Te	0.91	0.96	0.94	0.92	0.85	0.94	0.94	–
Afseth et al. (2010)	224	Nor	20-F CV	0.72	0.83	0.88	0.89	0.90	0.82	0.65	–
Coppa et al. (2010)	468	–	Tr/Te^b^	0.66	0.88	0.90	0.91	0.89	0.88	0.91	–
De Marchi et al. (2011)^a^	267	Bro	LOOCV	–	–	0.48	0.52	0.52	0.56	0.49	–
Soyeurt et al. (2011)	517	Mul	Tr/Te^b^	0.89	0.95	0.93	0.92	0.92	0.95	0.93	–
Ferrand et al. (2011)	250	Mul	Tr/Te	0.85	0.96	0.96	0.91	0.91	0.93	0.88	–
Eijndhoven et al. (2013)	1,236	Mul	Tr/Te^b^	0.92	0.93	0.92	0.93	0.85	0.95	0.93	–
Ferrand-Calmels et al. (2014)	345	Mul	R-Tr/Te	0.93	0.96	0.97	0.95	0.96	0.95	0.94	–
Lopez-Villalobos et al. (2014)	850	Cro	R-Tr/Te	0.73	0.78	0.81	0.81	0.86	0.77	0.74	0.33
Eskildsen et al. (2014)	890	Mul	10-F CV	–	0.88	0.89	0.91	0.91	0.90	0.91	0.63
Martin et al. (2015)^a^	422	–	20-F CV	0.82	–	–	–	–	0.82	0.66	–
Ferragina et al. (2015)^b^	1,264	Bro	R-Tr/Te^b^	–	–	–	0.67	–	–	0.60	–
Gottardo et al. (2016)	112	Mul	LOOCV	0.92	0.94	0.94	–	0.93	0.93	0.92	–
Bonfatti et al. (2016)	1,040	Sim	10-F CV	–	–	–	0.88	0.90	0.90	0.92	–
Fleming et al. (2017)	1,911	Mul	10-F CV	0.66	0.38	0.37	0.66	0.71	0.80	0.86	0.62
Ho et al. (2020)	240	Hol	10-F CV	0.94	0.94	0.90	0.89	0.90	0.93	0.95	–

^*a*^Correlation coefficient (r) transformed to coefficient of determination (R^2^). ^*b*^Bayes B methodology employed; multibreed (Mul); Norwegian Red (Nor); Brown Swiss (Bro); Crossbreed (Cro); Simmental (Sim); Holstein (Hol); number of folds (n-F) in the cross-validation, leave-one-out cross-validation (LOOCV), train and test cross-validation defined by splitting the data set randomly (R-Tr/Te) or not (Tr/Te), external or independent validation. *The validation strategy defined as “CV” was assigned for the reviewed paper that did not completely describe the validation method adopted or the authors defined that cross-validation was employed.

**TABLE 3:** Coefficient of determination in the validation set for the milk fatty acids predicted from mid-infrared in dairy cattle*.

References	C17:0	C18:0	C18:1^a^	C18:2^b^	C18:2^c^	C18:3^d^	SFA	MUFA	PUFA
Soyeurt et al. (2006)	–	0.69	–	0.07	0.62	0.14	0.94	0.85	0.39
Soyeurt et al. (2008)	–	0.85	–	–	–	–	–	0.93	
Rutten et al. (2009)	–	0.82	0.92	0.58	0.36	0.45	–	–	–
Afseth et al. (2010)	–	0.48	0.92	0.53	0.49	0.29	0.92	0.94	0.52
Coppa et al. (2010)	0.65	0.80	0.93	0.73	0.34	–	0.95	0.91	0.75
De Marchi et al. (2011)^1^	0.56	0.42	0.50	0.21	–	–	–	–	–
Soyeurt et al. (2011)	0.61	0.88	0.95	0.63	0.71	0.60	0.99	0.97	0.81
Ferrand et al. (2011)	–	0.77	0.91	0.70	0.65	–	0.98	0.92	0.38
Eijndhoven et al. (2013)	–	0.72	–	–	–	–	0.99		
Ferrand-Calmels et al. (2014)	–	0.85	0.97	0.83	0.78	–	1.00	0.98	0.78
Lopez-Villalobos et al. (2014)	0.43	0.60	0.87	0.64	0.66	0.51	0.93	–	0.73
Eskildsen et al. (2014)	0.54	0.82	0.82	0.37	0.65	–	–	–	–
Martin et al. (2015)^1^	–	0.62	0.84	–	–	–	0.77	0.86	–
Ferragina et al. (2015)^2^	–	0.49	–	–	–	–	–	–	–
Gottardo et al. (2016)	–	0.80	–	–	–	–	0.99	0.95	0.71
Bonfatti et al. (2016)	–	0.78	0.90	0.65	–	–	0.97	0.93	0.75
Fleming et al. (2017)	0.53	0.73	0.79	–	0.65	–	0.94	0.84	0.66
Ho et al. (2020)	0.82	0.81	0.72	–	–	–	–	–	–

^1^ Correlation coefficient (r) transformed to coefficient of determination (R^2^). ^2^ Bayes B methodology employed. ^a^ C18:1 cis9. ^b^ C18:2 cis9, trans 11. ^c^ C18:2 cis9, cis12. ^*d*^C18:3 cis9, cis12, cis15. SFA, saturate fatty acids; MUFA, monounsaturated fatty acids; PUFA, and polyunsaturated fatty acids. *The number of samples, breed, and validation strategy are described in Table 2.

For FA with less than 16 carbons (C4:0; C6:0; C8:0; C10:0; C12:0; and C14:0) the *R*^2^ reported varied from 0.37 to 0.97 in the validation set (Table 2). The feasibility of milk MIR spectra to predict FA with 16 carbons (C16:0 and C16:1) was investigated by several authors. The *R*^2^ observed in the validation sets were between the range of 0.33 and 0.95 (Table 2). For the FA with more than 16 carbons (C17:0; C18:0; C18:1 *cis*-9; C18:2 *cis*-9, *trans*-11; C18:2 *cis*-9, *cis*-12; and C18:3 *cis*-9, *cis*-12, *cis*-15) the *R*^2^ reported in the validation set varied from 0.07 to 0.95 (Table 3). The sums of saturated and monounsaturated FA were predicted with precision (*R*^2^) higher than 0.80, whereas for the polyunsaturated FA, the *R*^2^ varied from 0.38 to 0.81 in the validation set (Table 3). In general, FA with larger proportion (% of total FA) in the milk (e.g., C14:0, C16:0, and C18:1) presented greater *R*^2^ (e.g., C14:0, C16:0, and C18:1), when compared to milk FA of small individual proportion in milk (e.g., C17:0).

Studies have also investigated the effectiveness of using milk spectra data as a potential predictor of total protein, casein, and whey, as well as the individual caseins and whey proteins (Table 4). The *R*^2^ in the validation set for total protein and casein were greater than 0.70, except Sørensen et al. (2003); De Marchi et al. (2009), Rutten et al. (2011), and McDermott et al. (2016) that observed *R*^2^ in a range of 0.25–0.58. The *R*^2^ for total whey varied from 0.42 (McDermott et al., 2016) to 0.69 (Niero et al., 2016) in the validation set. For the individual caseins αS1-CN, αS2-CN, β-CN, and κ-CN *R*^2^ in a range of 0.18 (β-CN; Rutten et al., 2011) to 0.78 (αS1-CN; Ferrand et al., 2012) were reported in the validation set. The α-LA whey protein fraction presented the lowest prediction *R*^2^, varying from 0.06 (Eskildsen et al., 2016) to 0.48 (Ferrand et al., 2012), compared with β-LG where the prediction *R*^2^ were in a range of 0.34 (Eskildsen et al., 2016) to 0.64 (Bonfatti et al., 2011) in the validation set.

**TABLE 4:** Number of samples (N) and coefficient of determination in the validation set for the major protein content predicted from milk spectra using partial least square methodology in dairy cattle.

References	N	Breed	Validation	Prot	Cas	Whey	α_*S*__1_-CN	α_*S*__2_-CN	β-CN	κ-CN	α-LA	β-LG
Luginbühl (2002)	74	–	Tr/Te*	–	0.90	–	–	–	–	–	–	–
Sørensen et al. (2003)^a^	86	Multibreed	–	–	0.53	–	–	–	–	–	–	–
De Marchi et al. (2009)^a^	1,336	Simmental	20-F CV	0.58	0.58	0.53	0.50	0.35	0.32	0.43	0.29	0.55
Bonfatti et al. (2011)	1,517	Simmental	4-F CV	0.78	0.77	0.61	0.66	0.49	0.53	0.49	0.31	0.64
Rutten et al. (2011)	1,800	Holstein	R-Tr/Te	-	0.25	0.53	0.18	0.26	0.19	0.28	0.20	0.56
Ferrand et al. (2012)	193	Multibreed	Tr/Te	0.99	0.88	0.58	0.65	0.71	0.78	0.54	0.48	0.45
Bonfatti et al. (2016)	1,137	Simmental	10-F CV	0.81	0.80	0.53	0.74	0.49	0.58	0.39	0.24	0.48
Eskildsen et al. (2016)	832	Multibreed	Tr/Te	–	–	–	0.66	0.36	0.25	0.71	0.06	0.34
McDermott et al. (2016)^a^	730	Multibreed	4-F CV*	–	0.55	0.42	0.43	0.43	0.45	0.31	0.29	0.48
Niero et al. (2016)	114	Multibreed	LOOCV	0.88	0.88	0.69	–	–	0.60	0.74	0.37	0.47

Total protein (Prot), total casein (Cas), number of folds (n-F) in the cross-validation, leave-one-out cross-validation (LOOCV), train and test cross-validation defined by splitting the data set randomly (R-Tr/Te) or not (Tr/Te). ^*a*^Correlation coefficient (r) transformed to coefficient of determination (R^2^), and *external or independent validation.

The effectiveness of milk spectra data to predict mineral composition in dairy cattle has also been investigated. The prediction quality reported in the validation set for Ca, Mg, Na, and P is presented in Table 5. Model performances were satisfactory to predict Ca and P (*R*^2^ > 0.67) in Soyeurt et al. (2009); Toffanin et al. (2015), Visentin et al. (2016), and Franzoi et al. (2019). But the same minerals were poorly predicted (*R*^2^ < 0.55) by Gottardo et al. (2015); Bonfatti et al. (2016), Malacarne et al. (2018), and Fleming et al. (2019). For K, Mg, and Na the *R*^2^ reported by the authors varied from 0.25 (Malacarne et al., 2018) to 0.75 (Franzoi et al., 2019) in the validation set.

**TABLE 5:** Number of samples (N) and coefficient of determination in the validation set for mineral contents using partial least square methodology.

References	N	Breed	Validation*	Ca	K	Mg	Na	P
Soyeurt et al. (2009)	92	Multibreed	LOOCV	0.87	0.36	0.65	0.65	0.85
Gottardo et al. (2015)	208	–	10-F CV^b^	0.55	–	–	–	–
Toffanin et al. (2015)	208	Holstein	LOOCV	0.53^c^	–	–	–	0.70^c^
Bonfatti et al. (2016)	689	Simental	10-F CV	0.48	0.41	0.46	–	0.43
Visentin et al. (2016)	923	Multibreed	R-Tr/Te^b^	0.67	0.69	0.65	0.40	0.68
Malacarne et al. (2018)^a^	153	Holstein	Tr/Te^b^	0.25	0.34	0.26	0.25	0.53
Franzoi et al. (2019)^b^	93	Holstein	CV	0.79	0.55	0.68	0.75	0.87
Fleming et al. (2019)	986	Multibreed	10- FCV	0.25	–	–	–	–

^*a*^Bulk milk samples. ^*b*^Backward interval partial least squares (BiPLS), number of folds (n-F) in the cross-validation, leave-one-out cross-validation (LOOCV), train and test cross-validation defined by splitting the data set randomly (R-Tr/Te), external or independent validation. ^*c*^Correlation coefficient (r) transformed to coefficient of determination (R^2^). *The validation strategy defined as “CV” was assigned for the reviewed paper that did not completely describe the validation method adopted or the authors defined that cross-validation was employed.

Divergences in the predictability reported might be related to the different gold standard methodologies used in the reference data, population studied, and the sample size (De Marchi et al., 2014). Overall, the *R*^2^ observed in the published papers reviewed here highlight the potential of milk spectra data as a predictor of milk FA, proteins, and minerals. These studies also underline the need for future work using robust analytical data mining techniques and large datasets as a way to improve the model performance for phenotypes that have been inaccurate predicted. The possibility of more frequent predictions of such phenotypes could potentially create discoveries in different areas of animal science, such as genetics/genomics, nutrition, physiology, and reproduction. For example, some authors have demonstrated that milk fatty acids can be good predictors of plasma non-esterified fatty acids concentration (Mann et al., 2016; Dórea et al., 2017), which is an important phenotype associated with negative energy balance in lactating dairy cows. Additionally, such predictions can be used as a powerful tool to improve management decisions on livestock operations.

### Feed Intake

Given the economic impact of animal feed costs on farmer’s profitability, feed efficiency has been widely discussed as a key phenotype to be included in the selection indexes and for management decisions on livestock operations (Berry and Crowley, 2013; Berry, 2015; Seymour et al., 2019). Selecting animal for feed efficiency is highly attractive, but the practical implementation might be challenging, primarily because individual feed intake records on a large-scale are unavailable to date, and secondly some aspect of production, such as milk output or body weight, and energy sinks including maintenance, need to be accounted to determine individual feed efficiency (Berry and Crowley, 2013; Connor, 2015). Therefore, the use of NIR and MIR spectrometry has been explored as a potential tool to predict traits related to feed efficiency in beef and dairy cattle, as shown in Tables 6, 7. Seven studies evaluated the use of fecal NIR on fecal samples to predict organic or dry matter intake, reporting *R*^2^ ranging from 0.44 (Huntington et al., 2011) to 0.98 (Decruyenaere et al., 2004) in the validation set (Table 6). From the studies reviewed, only one study reported the use of feed (grass NIR) to predict dry matter intake, in which the *R*^2^ reported in the validation set was 0.71. It is important to point out that the results have not always been consistent, and few studies have compiled data sets of sufficient size to generate robust and accurate prediction equations. Furthermore, the use of NIR spectrometry on grab fecal and grass samples requires preparation and pretreatment, which is laborious, time-consuming, and may not be applicable on a large-scale.

**TABLE 6:** Number of samples (N), and coefficient of determination in the validation set (*R*^2^) for the prediction of dry matter intake (DMI) and organic matter intake (OMI) traits using grass near-infrared (G-NIR) and fecal near-infrared (F-NIR) spectrometry **^§^**.

References	N	Breed	Spectra	Trait	Validation	*R*^2^
Agnewa et al. (2004)	203	dairy	G-NIR	DMI	7-F CV	0.71
Boval et al. (2004)	88	beef	F-NIR	OMI	3-F CV	0.52
Decruyenaere et al. (2004)	139	dairy	F-NIR	DMI	CV	0.98
Garnsworthy and Unalt (2004)	91	dairy	F-NIR	DMI	R-Tr/Te^a^	0.97
Tran et al. (2010)	1,322	dairy	F-NIR	DMI	Tr/Te^a^	0.58
Huntington et al. (2011)	406	beef	F-NIR	DMI	CV	0.44
Landau et al. (2016)	125	beef	F-NIR	DMI	6-F CV	0.75
Johnson et al. (2017)	408	beef	F-NIR	DMI	CV	0.73

Number of folds (n-F) in the cross-validation (CV), train and test cross-validation defined by splitting the data set randomly (R-Tr/Te) or not (Tr/Te). ^*a*^External or independent validation. *The validation strategy defined as “CV” was assigned for the reviewed paper that did not completely describe the validation method adopted or the authors defined that cross-validation was employed. ^§^ All the studies applied partial least square statistical methodology.

**TABLE 7:** Number of samples (N) and coefficient of determination (*R*^2^) for dry matter intake (DMI), residual feed efficiency (RFI), effective energy intake (EEI), net energy intake (NEI), and energy intake (EI) traits using milk mid-infrared spectrometry in dairy cattle.

References	N	Breed	Trait	Method	Validation	*R*^2^
McParland et al. (2011)	5,469	Holstein	EEI	PLS	4-F CV*	0.74^a^
McParland et al. (2012)	4,109	Holstein	EEI	PLS	4-F CV*	0.64^a^
McParland et al. (2014)	1,335	Holstein	RFI	PLS	Tr/Te*	0.36^a^
McParland et al. (2014)	1,335	Holstein	EEI	PLS	Tr/Te*	0.49^a^
McParland et al. (2015)	1,270	Holstein	EI	PLS	20-F CV	0.56^*a*^
Shetty et al. (2017b)	1,044	Multibreed	DMI	PLS	R-Tr/Te*	0.77
Shetty et al. (2017b)	1,044	Multibreed	RFI	PLS	R-Tr/Te*	0.46
Dórea et al. (2018)	1,279	Holstein	DMI	ANN	LOOCV*	0.70
Wallén et al. (2018)	857	Norwegian red	DMI	PLS	5-F CV*	0.29^a^
Wallén et al. (2018)	857	Norwegian red	NEI	PLS	5-F CV*	0.42^a^
Lahart et al. (2019)	1,074	Multibreed	DMI	PLS	LOOCV*	0.64
Smith et al. (2019)	11,941	Holstein	EEI	PLS	4-F CV*	0.52
Grelet et al. (2020)	1,034	Holstein	DMI	SVM	R-Tr/Te	0.66

Number of folds (n-F) in the cross-validation, leave-one-out cross-validation (LOOCV), partial least square (PLS), artificial neural network (ANN), Support Vector Machine (SVM) train and test cross-validation defined by splitting the data set randomly (R-Tr/Te) or not (Tr/Te). *External or independent validation. ^*a*^Correlation coefficient (r) transformed to coefficient of determination R^2^.

Due to the difficulties in utilizing fecal or grass samples with NIR to predict intake, the value of milk MIR spectrometry for prediction of feed efficiency has also been evaluated (Table 7). As many milk recording schemes globally already use MIR spectrometry to predict protein, fat, casein, lactose, and urea contents (De Marchi et al., 2014), they can be a useful source of information on large-scale operations. The majority of researchers have aimed to predict dry matter intake, though some have also evaluated predictions for residual feed intake, net energy intake, and effective energy intake. For dry matter intake, the *R*^2^ in the validation set was in a range of 0.29 (Wallén et al., 2018) to 0.77 (Shetty et al., 2017b). The studies evaluating the use of milk spectra to predict effective energy intake reported *R*^2^ varying from 0.49 (McParland et al., 2014) to 0.74 (McParland et al., 2011). McParland et al. (2015) observed *R*^2^ of 0.56 for energy intake in the validation set. McParland et al. (2014) and Shetty et al. (2017b) reported *R*^2^ of 0.36 and 0.46, respectively, to predicted residual feed intake from MIR data in the validation set. The majority of the studies observed that combining MIR data with other animal-level variables, such as milk yield, body weight, and feeding behavior resulted in greater prediction precision and accuracy compared to predictions based only on MIR data. The reviewed studies suggest that milk MIR spectra data is a promising tool to predict indicator traits for feed efficiency in dairy cattle. Such a novel source of information has the potential to bring new insights into management decisions and breeding programs.

### Energy Balance

Dairy cows in early lactation are under high energy demand to meet their requirements for lactation and often energy intake is unable to meet a cow’s requirements, leading animals to enter in a period of negative energy balance (Collard et al., 2000; de Vries and Veerkamp, 2000; McParland et al., 2012). Effective and accurate early assessment of a cow’s energy balance could be useful for management strategies, mitigating the costs associated with detrimental effects of negative energy balance, and future genetic selection (McParland et al., 2012; Grelet et al., 2019). Energy balance has been estimated in dairy cows through alternative methods that are mostly based on the difference between energy intake and energy output or considering the change in body reserves (Coffey et al., 2001; Friggens et al., 2007; Banos and Coffey, 2010). The drawback to these methods is that they require regular measurements of energy intake, body condition score, and body weight, which are expensive to collect on a sufficiently large number of animals, not well suited to assess short-term changes, and vary with intake respectively (McParland et al., 2012). Moving forward to high throughput phenotyping, milk spectra have been used as a potential tool to predict energy balance (Table 8). McParland et al. (2011; 2012; 2014; 2015) reported *R*^2^ in a range of 0.29–0.56 in the validation set using evening milk spectra data. A moderate *R*^2^ (0.60) was reported by Smith et al. (2019) to predict energy balance in the validation set, whereas Ho et al. (2020) observed low *R*^2^ (0.48), which according to the authors could be due to the small dataset used when compared with the previous studies. The predictive ability observed by the authors highlights the potential of milk spectra data to predict herd or individual energy status level; however, more efforts are needed to improve the prediction quality. Furthermore, the models used only require milk spectra and yield, which are both routinely generated during milk recording. Therefore, farmers could have access to the individual animal energy status at the time of milking without additional cost.

**TABLE 8:** Number of samples (N) and coefficient of determination (*R*^2^) in the validation for energy balance trait using milk mid-infrared spectrometry in dairy cattle**^§^**.

References	N	Breed	Validation	*R*^2^
McParland et al. (2011)	5,469	Holstein	4-F CV*	0.56^a^
McParland et al. (2012)	4,109	Holstein	4-F CV*	0.29^a^
McParland et al. (2014)	1,335	Holstein	Tr/Te*	0.46^a^
McParland et al. (2015)	1,270	Holstein	20-F CV	0.53^a^
Ho et al. (2019)	240	Holstein	10-F CV	0.48
Smith et al. (2019)	11,941	Holstein	4-F CV*	0.60

Number of folds (n-F) in the cross-validation, train and test cross-validation defined by splitting the data set randomly (R-Tr/Te) or not (Tr/Te). *External or independent validation. ^*a*^Correlation coefficient (r) transformed to coefficient of determination (R^2^). ^§^All studies used partial least square methodology.

### Methane Emission

Strategies to predict enteric methane emission (CH_4_) have been widely explored by different research groups worldwide. Such interest is usually driven by concerns regarding the carbon footprint and lower feed efficiency due to energy losses in CH_4_ (Johnson and Johnson, 1995). Mitigating CH4 emissions may improve the livestock systems’ sustainability and profitability (Knapp et al., 2014). However, the majority of the classical methods used to quantify CH_4_ in the papers reviewed here (i.e., respiration chamber, sulfur hexafluoride tracer, and sniffer systems) are difficult, expensive, or not feasible to carry out on large scale operations (Hammond et al., 2016; Patra, 2016; Negussie et al., 2017). Several indirect measurements (e.g., feed intake, volatile fatty acids, milk FA, body weight, hindgut, feces, among others) have been proposed as a predictor of CH_4_ emission, in which milk FA have been stated as a promising CH_4_ proxy in dairy cattle (Negussie et al., 2017). Many studies have investigated the correlation between different milk FA, quantified using gas chromatography, and enteric CH_4_ production (Chilliard et al., 2009; Dijkstra et al., 2011; van Lingen et al., 2014; Rico et al., 2016). Based on the moderate to high correlations (0.50–0.80) observed by these authors, milk FA can be considered a potential indicator of individual animal enteric CH_4_ emissions. Since milk FA can be predicted from milk spectra, as previously discussed in section “Milk Composition,” several researchers have investigated the feasibility of using milk spectra data to predict the volume of CH_4_ eructed daily by a dairy cow (Table 9). Overall, the *R*^2^ reported by the papers reviewed here varied from 0.01 (Wang and Bovenhuis, 2019) to 0.79 (Dehareng et al., 2012) in the validation set. The variation in the predictive ability across studies can be partially explained by the different methods used to determine CH_4_ emission (e.g., respiration chambers, sulfur hexafluoride tracer, and sniffer method). Based on the prediction quality presented by some of the authors in experimental settings, milk spectra data has the potential to predict enteric CH_4_ emissions (Vanlierde et al., 2015). The validity of spectra data to predict CH_4_ emissions under conditions more similar to a commercial herd was only partly confirmed only by Shetty et al. (2017a). In beef cattle, the majority of studies have measured methane emission using classical direct methods, but to the best of our knowledge, no studies have attempted to predict methane emission indirectly using infrared spectrometry data.

**TABLE 9:** Number of samples (N) and coefficient of determination in validation set (*R*^2^) for methane emission trait predicted from milk mid-infrared spectra data.

References	N	Breed	Method	Validation	*R*^2^
Dehareng et al. (2012)	60	Holstein	PLS	LOOCV	0.79
Vanlierde et al. (2015)	446	Multibreed	PLS	Tr/Te*	0.23^a^
Vanlierde et al. (2016)	532	Multibreed	PLS	5-F CV	0.70
Shetty et al. (2017a)	2,202	Holstein	PLS	R-Tr/Te*	0.39
Bittante and Cipolat-Gotet (2018)	1,150	Brown Swiss	Bayes B	R-Tr/Te	0.57
Vanlierde et al. (2018)	584	Multibreed	PLS	5-F CV	0.57
van Gastelen et al. (2018)	218	Holstein	PLS	10-F CV	0.49
Wang and Bovenhuis (2019)	801	Holstein	PLS	LOOCV*	0.01

Partial least square (PLS), multivariate linear regression (MLR), number of folds (n-F) in the cross-validation (CV), leave-one-out cross-validation (LOOCV), train and test cross-validation defined by splitting the data set randomly (R-Tr/Te) or not (Tr/Te). *External validation. ^*a*^Correlation coefficient (r) transformed to coefficient of determination (R^2^).

### Fertility

Although fertility is a non-yield trait, it is the key to overall profitability in cattle farming as poor fertility increases the replacement rate due to involuntary culling, costs related to fertility treatments, and multiple inseminations, which directly affect animal production (Boichard, 1990; Dekkers, 1991; González-Recio et al., 2004; Berry et al., 2014; Pravia et al., 2014; Kaniyamattam et al., 2016). Since fertility traits are difficult and expensive to measure, early indicator or associated traits (e.g., body condition score, body weight, metabolic and endocrine blood traits, and milk composition) can be used either to enhance indirect genetic improvement of fertility or for reproductive management decisions (Berry et al., 2003; Moraes et al., 2007; Friggens et al., 2008; Diskin and Kenny, 2014). Hempstalk et al. (2015) developed a biological-based ML model to predict the likelihood of conception success given the herd- and cow-specific attributes, with particular attention to the use of milk spectral data. The area under the curve reported by the authors in the external validation set varied from 0.49 to 0.60 across different ML algorithms. However, the inclusion of milk spectra, compared with the same model using only non-MIR data (e.g., days in milk, milk yield, number of inseminations, breeding values, among others) in the ML models, did not improve the accuracy of predicting the likelihood of conception to an insemination. The prediction accuracies of pregnancy status using milk spectra data were also assessed by Toledo-Alvarado et al. (2018). The area under the curve across breed had similar patterns averaging 0.61 for Holsteins and 0.64 for Alpine Grey cows in the cross-validation. The authors concluded that pregnant versus open cows post insemination could be discriminated with promising accuracy using milk spectra, parity, and days in milk. Ho et al. (2019) reported that milk spectra from early lactation cow together with other on-farm data (e.g., days in milk, days from calving to insemination, calving age, milk yield, genotypes, among others) could be used to classify cows that conceived at first insemination or did not conceive within the breading season with reasonable accuracy, based on the area under the curve (0.75), in herd-by-herd external validation. Delhez et al. (2020) observed that milk spectra recorded after 150 days of pregnancy was promising to predict the pregnancy status in Holstein, with the area under the curve around 0.76 in cow-independent external validation. More efforts need to be made to investigate the reliability of milk spectra to predict fertility traits since the accuracies observed to date are not high and the number of studies is very low, although recent. Nevertheless, these studies provide new insights into novel phenotypes that can be used indirectly to improve fertility, especially in dairy cattle, which could become an important tool for management decisions on dairy farms.

### Health Status

Several metabolic disorders and diseases, such as ketosis, mastitis, milk fever, lameness, displaced abomasum, metritis, retained placenta, and cystic ovaries have important impacts on profitability and animal welfare (Kelton et al., 1998; Friggens et al., 2007; McArt et al., 2015; Jamrozik et al., 2016). To mitigate herd losses, producer-recorded events have been used for management decisions at farmer-level and genetic selection (Jamrozik et al., 2013; Miglior et al., 2014; Luke et al., 2019). However, the bottleneck relies on the difficulty of routinely collecting high-quality direct phenotypes on farms (Egger-Danner et al., 2015). Subclinical hyperketonemia or ketosis is one of the most frequent diseases in dairy cattle and it is characterized by increased concentrations of the ketone bodies acetoacetate, β-hydroxybutyrate (BHB), and acetone in blood, milk, and urine (Hansen, 1999). Additionally, blood metabolites such as glucose, non-esterified fatty acids (NEFA), blood urea nitrogen (BUN), and insulin-like growth factor 1 (IGF-1), and glutamic oxaloacetic transaminase (GOT) might also be used as indicators of metabolic status in dairy cows (Fenwick et al., 2008; Benedet et al., 2019; Grelet et al., 2019). Blood metabolic profile testing is the gold standard for diagnosis, however, it is invasive, logistically challenging, and costly (Luke et al., 2019). MIR spectrometry has been explored as possible high-throughput phenotyping technology to predict BHB concentration in blood or milk, and acetone in milk (Table 10). Within the published papers de Roos et al. (2007) and Grelet et al. (2016) predicted the concentration of BHB in milk and the *R*^2^ reported by the authors in the validation was 0.62 and 0.63, respectively. Seven published papers evaluated the use of milk spectra as a predictor of BHB in serum and the *R*^2^ varied from 0.40 (Belay et al., 2017) to 0.70 (Grelet et al., 2019) in the validation set. Although few studies have focused on predicting acetone in milk from milk spectra, the *R*^2^ observed by Hansen (1999) and de Roos et al. (2007) were higher than 0.70, except Grelet et al. (2016) which observed *R*^2^ of 0.67 in the validation set. Heuer et al. (2001) observed a standard error of cross-validation, the prediction quality metric used by the authors, of 0.24 to predict acetone in milk. Likewise, the feasibility of using spectral data to predict glucose, NEFA, BUN, and IGF-1 were also investigated in this review (Table 10). The *R*^2^ reported by the authors varied between 0.20 (glucose; Benedet et al., 2019) to 0.61 (IGF-1; Grelet et al., 2019) in the validation set.

**TABLE 10:** Number of samples (N) and coefficient of determination (*R*^2^) in the validation set for β-hydroxybutyrate (BHB), acetone (Ac), non-esterified fatty acids (NEFA), blood urea nitrogen (BUN), glucose (Glu), glutamic oxaloacetic transaminase (GOT), and insuline-like growth factor 1 (IGF-1) using milk mid-infrared spectrometry in dairy cattle.

References	N	Breed	Sample	Trait	Method	Validation*	*R*^2^
Hansen (1999)	310	–	Milk	Ac	PLS	Tr/Te	0.81
Heuer et al. (2001)	180	–	Milk	Ac	PLS	LOOCV	0.24^b^
de Roos et al. (2007)	1,080	Holstein	Milk	Ac	PLS	CV	0.72^c^
de Roos et al. (2007)	1,080	Holstein	Milk	BHB	PLS	CV	0.62^c^
Grelet et al. (2016)	224	Holstein	Milk	Ac	PLS	R-Tr/Te^a^	0.67
Grelet et al. (2016)	434	Holstein	Milk	BHB	PLS	R-Tr/Te^a^	0.63
Belay et al. (2017)	1,914	Holstein	Blood	BHB	PLS	R-Tr/Te^a^	0.40
Pralle et al. (2018)	3,629	Holstein	Blood	BHB	ANN	R-Tr/Te^a^	0.56
Bonfatti et al. (2019)	1,910	Multibreed	Blood	BHB	PLS	R-Tr/Te^a^	0.52
Benedet et al. (2019)	295	Multibreed	Blood	BHB	PLS	3-F CV	0.63
Benedet et al. (2019)	294	Multibreed	Blood	NEFA	PLS	3-F CV	0.52
Benedet et al. (2019)	294	Multibreed	Blood	BUN	PLS	3-F CV	0.58
Benedet et al. (2019)	294	Multibreed	Blood	Glu	PLS	3-F CV	0.20
Benedet et al. (2019)	294	Multibreed	Blood	GOT	PLS	3-F CV	0.24
Grelet et al. (2019)	205	Holstein	Blood	BHB	PLS	4-F CV	0.70
Grelet et al. (2019)	234	Holstein	Blood	NEFA	PLS	4-F CV	0.39
Grelet et al. (2019)	387	Holstein	Blood	IGF-1	PLS	4-F CV	0.61
Grelet et al. (2019)	380	Holstein	Blood	Glu	PLS	4-F CV	0.44
Luke et al. (2019)	878	Holstein	Blood	BHB	PLS	R-Tr/Te^a^	0.60
Luke et al. (2019)	878	Holstein	Blood	NEFA	PLS	R-Tr/Te^a^	0.45
Luke et al. (2019)	878	Holstein	Blood	BUN	PLS	R-Tr/Te^a^	0.35
Müller et al. (2019)	585	Holstein	Blood	BHB	PLS	CV	0.42

Partial least square (PLS), artificial neural network (ANN), train and test cross-validation defined by splitting the data set randomly (R-Tr/Te), number of folds (n-F) in the cross-validation (CV), leave-one-out cross-validation (LOOCV). ^*a*^External or independent validation. ^*b*^Standard error in the cross-validation as accuracy metric. ^*c*^Correlation coefficient (r) transformed to coefficient of determination (R^2^). *The validation strategy defined as “CV” was assigned for the reviewed paper that did not completely describe the validation method adopted or the authors defined that cross-validation was employed.

Mastitis is the most common and costly contagious disease in dairy cattle characterized as an inflammation of the mammary gland and udder tissue. To the best of our knowledge, only Rienesl et al. (2019) investigated the possibility of using milk spectra to predict mastitis, which reported satisfactory accuracy (0.68) in the validation data set (Table 11). Mineur et al. (2017) and Bonfatti et al. (2020) investigated the ability of milk spectra data as a predictor of lameness and the predictions were poor to be employed as an on-farm tool to detect lameness in cows (Table 11). Based on the results presented by the reviewed papers, milk spectra might be useful to predict the concentration of BHB in serum or milk, acetone on milk, and mastitis occurrence in dairy cattle. However, more studies using larger and more diverse calibration data sets are needed, especially across countries, to improve the prediction quality before models can be used for on-farm management or genetic selection purposes.

**TABLE 11:** Number of samples (N), accuracy (Acc), sensitive (Sen), and specificity (Spe) in the validation set for mastitis (Mas) and lameness (Lam) traits using milk mid-infrared spectrometry using partial least square in dairy cattle.

Reference	N	Breed	Trait	Validation	Acc (%)	Sen (%)	Spe (%)
Mineur et al. (2017)	9,811	Multibreed	Lam	R-Tr/Te	–	60	62
Rienesl et al. (2019)	2,340	Multibreed	Mas	R-Tr/Te*	68	57	79
Bonfatti et al. (2020)	3,771	Multibreed	Lam	10-F CV	62	57	62

Train and test cross-validation defined by splitting the data set randomly (R-Tr/Te), number of folds (n-F) in the cross-validation (CV), and *external or independent validation.

### Meat Traits

Meat quality is a complex concept that involves many attributes such as tenderness, juiciness, flavor, marbling, color, and shelf life (Williams, 2008). Meat tenderness is one of the most important attributes affecting consumers’ acceptability, followed by fat content and visual attributes (Shackelford et al., 2001; Liu et al., 2003; Williams, 2008). Considering the increasing demands for meat and consumers willing to pay higher prices for certified, high-quality meat products, there is a growing interest by the beef meat chain to accurately assess meat quality traits (Andrés et al., 2008; Prieto et al., 2008). To date, meat quality traits have been measured using physical methods, which are time-consuming, expensive, destructive (depreciating the value of the carcass), and unsuitable to perform individually in large-scale (Su et al., 2018; Chapman et al., 2019). To satisfy the requirements of the modern meat industry, NIR spectrometry has been stated as an alternative tool for high throughput phenotyping meat quality traits because it is considered an accurate, fast, non-invasive and non-destructive technique with great potential for in-line application (Prevolnik et al., 2004; Prieto et al., 2009a; Chapman et al., 2019). The feasibility and robustness of NIR technique to predict meat quality traits in cattle have been investigated by several researchers (Tables 12–14). Here our focus will be on meat tenderness, intramuscular fat content, meat color, and cooking loss traits, since such attributes impact consumers’ satisfaction. Meat quality traits can be measured using different methodologies, but in our review, such traits were summarized regardless of the methodology applied. The *R*^2^ observed in the reviewed papers for meat tenderness varied from 0.12 (De Marchi et al., 2007) to 0.81 (Prieto et al., 2014) in the validation set (Table 12). The *R*^2^ for intramuscular fat content varied from 0.02 (Magalhães et al., 2018) to 0.99 (Su et al., 2014) in the validation set (Table 13). For color traits (L^∗^, a^∗^, and b^∗^), the *R*^2^ in the validation set (Table 14) were in a range of 0.16 (Magalhães et al., 2018) to 0.93 (Zhang et al., 2015). The *R*^2^ observed for cooking losses varied from 0.001 (Prieto et al., 2008) to 0.61 (Zhang et al., 2015) in the validation set (Table 14). Overall, NIR spectrometry has shown great potential to assess meat quality traits within different breeds. Furthermore, meat quality traits can be generated directly on the raw beef under slaughterhouses conditions as the NIR technique may not require sample pre-preparation. Nevertheless, further research needs to be conducted to validate the models across breeds and use modern data mining approaches to improve prediction quality.

**TABLE 12:** Number of samples (N) and coefficient of determination (*R*^2^) in the validation set for meat tenderness trait predicted from near-infrared spectrometry in cattle.

References	N	Breed	Method	Validation^∗^	*R*^2^
Mitsumoto et al. (1991)	11	Japanese Black	MLR	–	0.67^b^
Hildrum et al. (1994)	10	Norwegian	PCR	CV	0.29^b^
Byrne et al. (1998)	70	–	PLS	CV	0.37^b^
Park et al. (1998)	119	–	PLS	Tr/Te	0.63
Rødbotten et al. (2000)	79	Norwegian Red	PLS	LOOCV	0.36^b^
Rødbotten et al. (2001)	48	Norwegian	PLS	CV	0.72^b^
Venel et al. (2001)	67	–	PLS	LOOCV	0.31^b^
Leroy et al. (2003)	189	Belgian White Blue	PLS	CV	0.25
Liu et al. (2003)	22	Multibreed	PLS	LOOCV	0.48
Shackelford et al. (2005)	146	Multibreed	MLR	Tr/Te	0.22
De Marchi et al. (2007)	148	Piamontese	PLS	4-FCV	0.12
Andrés et al. (2008)	112	Maronesa	PLS	LOOCV	0.53
Ripoll et al. (2008)	190	Multibreed	PLS	R-Tr/Te	0.74
Prieto et al. (2008)	67	-	PLS	LOOCV	0.17
Prieto et al. (2009b)	194	Crossbred	PLS	LOOCV	0.31
Rosenvold et al. (2009)	381	Hereford	PLS	R-Tr/Te	0.58
Cecchinato et al. (2009)	1,298	Piamontese	PLS	Tr/Te	0.50
Yancey et al. (2010)	40	Multibreed	PLS	LOOCV	0.28^b^
Cecchinato et al. (2011)	1,208	Piamontese	PLS	R-Tr/Te	0.21
De Marchi et al. (2013)	336	Multibreed	PLS	8-FCV	0.34
De Marchi (2013)	81	Crossbred	PLS	LOOCV	0.13
Prieto et al. (2014)	63	Crossbred	PLS	LOOCV	0.81
Zhang et al. (2015)	162	Yak	PLS	R-Tr/Te	0.43
Magalhães et al. (2018)	644	Nelore	PLS	LOOCV	0.40
Su et al. (2018)	442	Multibreed	PLS	R-Tr/Te^a^	0.60
Qiao et al. (2015)	234	-	SVM	Tr/Te	0.20
Wyrwisz et al. (2019)	89	Holstein	PLS	TR/Te	0.62
Savoia et al. (2020)	1,166	Piamontese	Bayes B	LOOCV^a^	0.16
Cafferky et al. (2020)	595	Multibreed	PLS	LOOCV	0.22

Mulitple linear regression (MLR), principal components regression (PCR), partial least square (PLS), support vector machine (SVM), number of folds (n-F) in the cross-validation (CV), leave-one-out cross-validation (LOOCV), train and test cross-validation defined by splitting the data set randomly (R-Tr/Te) or not (Tr/Te). ^*a*^External or independent validation. ^*b*^Correlation coefficient (r) transformed to coefficient of determination (R^2^). *The validation strategy defined as “CV” was assigned for the reviewed paper that did not completely describe the validation method adopted or the authors defined that cross-validation was employed.

**TABLE 13:** Number of samples (N) and coefficient of determination (*R*^2^) in the validation set for intramuscular fat content predicted from near-infrared spectrometry in cattle.

References	N	Breed	Method	Validation*	*R*^2^
Mitsumoto et al. (1991)	11	Japanese Black	MLR	–	0.92^2^
Sanderson et al. (1997)	72	British Friesian	PLS	4-FCV	0.95
Rødbotten et al. (2000)	79	Norwegian Red	PLS	LOOCV	0.58^b^
Cozzolino and Murray (2002)	100	–	PLS	4-FCV	0.86
Cozzolino et al. (2002)	78	Hereford	PLS	4-FCV	0.92
Prevolnik et al. (2005)	34	Multibreed	PLS	CV	0.93
Ripoll et al. (2008)	190	Multibreed	PLS	R-Tr/Te^a^	0.76
Prieto et al. (2011)	194	Multibreed	PLS	LOOCV	0.43
Cecchinato et al. (2012)	148	Piamontese	PLS	4-FCV	0.82
Prieto et al. (2014)	63	Crossbred	PLS	LOOCV	0.86
Su et al. (2014)	182	Multibreed	PLS	R-Tr/Te^a^	0.99
Dixit et al. (2016)	108	–	PLS	Tr/Te	0.82
Magalhães et al. (2018)	644	Nelore	PLS	LOOCV	0.02

Mulitple linear regression (MLR), partial least square (PLS), number of folds (n-F) in the cross-validation (CV), leave-one-out cross-validation (LOOCV), train and test cross-validation set defined by splitting the data randomly (R-Tr/Te) or not (Tr/Te). ^*a*^External or independent validation. ^*b*^Correlation coefficient (r) transformed to coefficient of determination (R^2^). *The validation strategy defined as “CV” was assigned for the reviewed paper that did not completely describe the validation method adopted or the authors defined that cross-validation was employed.

**TABLE 14:** Number of samples (N) and coefficient of determination (*R*^2^) in the validation set for L^∗^ (*R*^2^_L*_), a^∗^ (*R*^2^_a*_), and b^∗^ (*R*^2^_b*_) meat color, and cooking losses (*R*^2^_CL_) traits predicted from near-infrared spectrometry in cattle.

References	N	Breed	Method	Validation^§^	*R*^2^_L*_	*R*^2^_a*_	*R*^2^_b*_	*R*^2^_CL_
Mitsumoto et al. (1991)	11	Japanese black	MLR	CV	–	–	–	0.59^*b*^
Leroy et al. (2003)	189	Belgian White Blue	PLS	CV	0.83	0.39	0.75	0.25
Liu et al. (2003)	113	Multibreed	PLS	LOOCV	0.55	0.90	0.78	–
De Marchi et al. (2007)	148	Piamontese	PLS	4-FCV	–	–	–	0.15
Andrés et al. (2008)	109	Maronesa	PLS	LOOCV	0.80	0.23	0.27	0.02
Prieto et al. (2008)	67	–	PLS	LOOCV	0.87	0.71	0.90	0.001
Prieto et al. (2009b)	194	Crossbred	PLS	LOOCV	0.83	0.76	0.84	0.23
Cecchinato et al. (2009)	1,298	Piamontese	PLS	CV	0.65	0.69	0.81	0.50
Cecchinato et al. (2011)	1,208	Piamontese	PLS	R-Tr/Te	0.64	0.68	0.44	0.04
De Marchi et al. (2013)	336	Multibreed	PLS	8-FCV	0.70	0.73	0.60	0.38
De Marchi (2013)	81	Crossbred	PLS	LOOCV	0.41	0.58	0.57	0.31
Prieto et al. (2014)	63	Crossbred	PLS	LOOCV	0.80	0.71	0.77	–
Zhang et al. (2015)	162	Yak	PLS	R-Tr/Te	0.74	0.81	0.93	0.61
Qiao et al. (2015)	234	–	SVM	Tr/Te	0.80	0.64	0.54	–
Magalhães et al. (2018)	644	Nelore	PLS	LOOCV	0.16	0.17	0.45	–
Su et al. (2018)	442	Multibreed	PLS	R-Tr/Te^*a*^	0.61	0.64	0.38	0.56
Wyrwisz et al. (2019)	89	Holstein	PLS	Tr/Te	0.33	0.57	0.61	0.47
Savoia et al. (2020)	1,166	Piamontese	Bayes B	LOOCV^*a*^	0.84	0.55	0.63	0.16

*Mulitple linear regression (MLR), partial least square (PLS), support vector machine (SVM), number of folds (n-F) in the cross-validation (CV), leave-one-out cross-validation (LOOCV), train and test cross-validation set defined by splitting the data randomly (R-Tr/Te) or not (Tr/Te). ^*a*^External or independent validation. ^2^Correlation coefficient (r) transformed to coefficient of determination (R^2^). ^§^The validation strategy defined as “CV” was assigned for the reviewed paper that did not completely describe the validation method adopted or the authors defined that cross-validation was employed.*

## Data Mining

Frequently, the main goal of using infrared spectrometry technology in the livestock industry is the development of predictive models to determine the content of specific compounds present in products such as milk, meat, and feedstuffs. However, many compounds present in such products are highly correlated with phenotypes that are difficult to measure in commercial and research settings, such as feed intake, methane emission, energy balance, methane emission, fertility, metabolic diseases, and meat quality traits, as previously discussed. In this context, several research studies have attempted to develop predictive models to predict such complex phenotypes for management decisions or breeding purposes. However, we have noted that factors such as the analytical method chosen to develop the predictive models and the cross-validation strategy used to evaluate the analytical approaches are not deeply discussed in the research studies involving livestock data. If not utilized properly, those two factors can result in (a) poor predictions due to the lack of ability of certain models to capture complex relationships between explanatory and response variables and (b) overoptimistic prediction quality due to high data dependency occurring between training and validation dataset. To developed robust predictive models using spectral data the following three steps should be followed: (1) spectra pretreatment, to remove noise or non-informative wavenumbers, (2) model training (or algorithm training), in which analytical techniques are used to assess the set of coefficients, number of latent variables, or hyperparameters, and (3) model validation, in which an independent dataset is used to evaluate the predictive ability of the model developed using the training dataset. These three main steps will be discussed throughout this review section.

### Spectra Preprocessing

Infrared spectra data comprise signals related to compounds present in the biological sample as well as non-informative signals coming from background, high-frequency noise, baseline shift, and overlapping bands (Rinnan et al., 2009a). Therefore, preprocessing spectral data is a common and crucial strategy that helps to mitigate such undesirable signals present in the raw data, maximizing the relationship between the infrared spectrum and the target phenotype (Rinnan et al., 2009a; De Marchi et al., 2014; McParland and Berry, 2016). Furthermore, preprocessing the spectra data prior to fit the calibration model is used in attempting to obtain robust prediction models and to restrict the insertion of bias into the model. However, applying unsuitable or a high stringent preprocessing strategy might remove important information from the biological sample (Rinnan et al., 2009a). Spectra preprocessing are commonly performed using mathematical pretreatment techniques or variable selection approach. The main mathematical pretreatment techniques used in the reviewed papers to mitigate signal noise can be divided into two groups, scatter-correction methods (e.g., multiplicative scatter correction, standard normal variate, and orthogonal scatter correction) and spectral derivatives (e.g., Savitzky-Golay polynomial derivative). Multiplicative scatter correction (MSC) is used to remove physical effects including particle size and surface blaze from the spectra, which do not carry any chemical or physical information, by correcting differences in the baseline and the trend (Martens et al., 1983). Standard normal variate (SNV) aims to remove the multiplicative effects of scatter and particle size, giving the sample a unit standard deviation (Barnes et al., 1989). Orthogonal scatter correction (OSC) eliminates the parts linearly unrelated (orthogonal) to the response variable (Wold et al., 1998). Savitzky-Golay (SG) first derivative is used to improve the spectra resolution by eliminating constant baseline, whereas the second derivative eliminates both baseline and linear trend (Savitzky and Golay, 1964). More details and theory overview about the mathematical pretreatment techniques employed on NIR and MIR spectra can be found in Rinnan et al. (2009a, b). From the 113 published papers reviewed here, researchers have used spectra data without pretreatment (50), SG first derivative (28), SG second derivative (10), MSC (7), SNV (5), and OSC (2). Eleven authors did not report if some mathematical pretreatment was applied to the spectra data. Several authors also used the combination of more than one pretreatment strategy (e.g., SG first derivative + MSC; SG second derivative + SNV; and SG first derivative + SG second derivative + MSC). Some authors have reported an increase in the prediction quality using pretreated spectra data (Heuer et al., 2001; Rødbotten et al., 2001; Soyeurt et al., 2011; De Marchi et al., 2011), whereas others have observed that the pretreatment failed to improve prediction accuracies (McParland et al., 2011; Cecchinato et al., 2012; Dehareng et al., 2012; Shetty et al., 2017a; Cafferky et al., 2020).

Analytical methods commonly employed on spectra data are suitable to reduce the full-spectrum to a few latent variables or perform some type of regularization (e.g., ridge and lasso). However, even performing such dimensionality reduction, they may also be penalized by noise or non-informative wavenumbers. Furthermore, the number of wavenumbers in infrared spectrometry datasets usually outnumbers the sample size. For example, a dataset containing 30 samples with their respective spectra data (1,060 wavenumbers). Note that the number of parameters *p* (1,060 wavenumbers) is greater than the number of observations *n* (30 samples). In such situations, the use of least squares is not appropriate since it will yield a set of coefficient estimates that result in a perfect fit to the data and the residuals will be zero (James et al., 2013). This phenomenon is a concern in data analysis because such perfect fit will potentially lead to overfitting the data (Hastie et al., 2009). Variable selection, therefore, is an important strategy used prior to the model calibration to reduce the data dimensionality by selecting a subset of relevant features from the original space to improve the model robustness and reduce the model complexity (Koljonen et al., 2008; Rinnan et al., 2009a). Although several authors removed the water noise wavenumbers, here we are only taking into account studies that performed variable selection based on a mathematical or statistical approach. Thus, only nine out of 113 published papers reviewed here performed variable selection prior to the calibration model. Variable selection was employed using a genetic algorithm (GA), uninformative variable elimination (UVE), variable importance for projection (VIP), and coefficient of variation (CV) combined with Markov Blanket (MB) techniques by 4, 3, 2, and 1 studies, respectively. Briefly, GA is an algorithm based on the biological evolution theory and natural selection and the main idea is to find within the set of predictor variables the ones that best fitted the model. The best predictor variables need to show high “fitness” and probability to “survive” to be included in the subsequent variable sets used for model refit. An iterative process is performed until the GA has selected the best predictor variable set or the best combination of them (Leardi et al., 1992). UVE approach adds artificial noise predictor variables, multiplied by a constant close to zero in order to eliminate any possible interaction with the original variables, to the reference dataset before fitting the model. The wavenumbers (original variables) that play a less important role in the model (based on the root mean square error, for example) than the random variables are considered uninformative and eliminated from the dataset before the procedure is repeated. The iterative process is performed until the stop criterion is reached (Centner and Massart, 1996). Using the VIP technique, one calculates a coefficient *v* that represents the importance or influence of each predictor variable on the response variable. Thus, predictor variables with a *v* < user-defined threshold *u* (*v* < 1, for example) are less relevant to fit the model and can be eliminated (Wold et al., 1993). CV is a well-known standardized measure of dispersion and it is used to eliminate wavenumbers that lack variability between samples. The MB notion is widely used in Bayesian Network and is defined as: for a node (target variable), its MB is the minimal set of parents, children, and spouses (wavenumbers) that best represents the node (Pearl, 1988). Studies that performed variable selection prior to the calibration step have reported an improvement in the quality of the predictions for different phenotypes and analytical approaches (Wu et al., 2009, 2012; Gottardo et al., 2015, 2016; Niero et al., 2016; Dórea et al., 2018). Although the authors have observed that spectra pretreatment and variable selection improved the predictions accuracies, there is no consensus in the literature regarding which situations such techniques will effectively result in better prediction ability, especially using larger datasets. Therefore, the effect of spectra data pretreatment and variable selection on the prediction quality should be more deeply investigated. Furthermore, as pointed out by De Marchi et al. (2014) and observed in the published papers reviewed here, the authors usually report only the precision and accuracy for the best model, while information regarding other models is not shown and discussed for full comparison.

### Calibration Models

From the 113 published papers retrieved from Web of Science, Partial Least Squares (PLS) was the most used statistical approach (101 papers) for the development of predictive equations, when compared to other machine learning (ML) methods. The simple implementation makes PLS a well-established and widely used methodology to generate novel complex traits from infrared spectrometry data in different fields. PLS is a dimension reduction technique suitable when the number of predictors is greater than the number of observations (e.g., infrared spectra data) as well as when strong collinearity exists between predictor variables, i.e., some wavenumbers can be rewritten as a linear function of others (Martens and Naes, 1987). Briefly, PLS maximizes the covariance between the predictor variables and the response variable resulting in a small set of components (latent variables), commonly called factors, that are used to predict target phenotypes in a new dataset (Martens and Jensen, 1982; Martens and Naes, 1987). Multiple linear regression (MLR) has also been used (Mitsumoto et al., 1991; Shackelford et al., 2005) or compared to other methodology (Pralle et al., 2018; Müller et al., 2019) to predict complex phenotypes using spectra data. MLR solves a number of simultaneous equations exploring the linear relationship between several explanatory variables (i.e., wavenumbers) and the continuous response variable (Hastie et al., 2009). Despite the power of such statistical method, Pralle et al. (2018) did not observe improvement in the prediction quality. In contrast, Müller et al. (2019) reported improvement when MLR was implemented compared with other methodologies such as PLS. In addition, the bottleneck of MLR methodology is that its implementation will often face the problem that the number of parameters is greater than the number of samples (Hastie et al., 2009). The PLS method to date has succeeded in predicting some complex traits with high accuracies, whereas for other traits, the prediction quality was poor, as previously reported in section “Complex Traits Predicted by Infrared Spectrometry Data.” This fact highlights the need for more research studies evaluating other analytical strategies able to deal with missing data, non-linear relationships between response and explanatory variables, and high-dimensional data to improve the prediction quality of complex phenotypes. Recently, Bayesian and ML techniques have been implemented to predict a range of phenotypes in livestock; however, it is at a small proportion compared with the number of studies using PLS. Bayesian models have been developed for high-dimensional regression and they are widely used in the context of genomic prediction (Meuwissen et al., 2001). In addition to the possibility of assigning prior information for the marker effects, here substituted by the wavenumbers’ effects, Bayesian methods are also able to perform estimate shrinkage and variable selection. For genomic prediction, Bayesian methods may have greater predictive power than dimension-reduction methods (Meuwissen et al., 2001; de los Campos et al., 2013). Ferragina et al. (2015) reported that Bayes B approach, which is one of the many Bayesian methods available, outperformed PLS methods for predicting milk components and technological properties using infrared spectral data and Bayes B was able to select a small subset of important wavenumbers. Based on Ferragina et al. (2015) finds, Bittante and Cipolat-Gotet (2018); Toledo-Alvarado et al. (2018), and Savoia et al. (2020) also used Bayes B methodology to predict complex phenotypes using spectra data. ML techniques such as Support Vector Machine (SVM) and Artificial Neural Networks (ANN) have been tested as an alternative to the traditional PLS method, because of their ability to search in a high-dimensional space of predictor variables for features that best describe the response variable, with the ability to self-learn. Additionally, such methods can better model the complex relationships (e.g., non-linear and interactions) between the input variables and the response outcome, which could improve the prediction quality (Gianola et al., 2011). Indeed, the authors that used SVM or ANN approaches have reported an increase in prediction quality compared with PLS method (Hempstalk et al., 2015; Qiao et al., 2015; Dórea et al., 2018; Pralle et al., 2018; Grelet et al., 2020). Although ML methodologies are suitable to be implemented in the high-dimensional space of predictor variables, this approach tends to easily overfit, in general because of small datasets and noisy data (Hempstalk et al., 2015). Overfitting is a recurrent issue in ML methods, such as in ANN, and it is clearly identified by high prediction accuracy in the training dataset but very poor in the validation dataset. For such methods, it is very important to perform unbiased validation strategy, therefore ensuring maximum possible data independency between training and validation would be ideal to unbiased predictions (Roberts et al., 2017). Additionally, ML techniques often require an extensive search for hyperparameters (e.g., number of neurons, number of layers, learning rate, among others) before training the algorithms to perform final prediction (Bengio, 2012). Such search is critical in order to define the network architecture, which may detrimentally affect prediction quality if not appropriate (Bengio, 2012). Based on the comparisons between traditional (PLS) and advanced analytical methods (Bayesian and ML) found in the reviewed papers, the use of advanced techniques resulted in improved prediction accuracy for some complex phenotypes. However, the prediction quality is influenced by many factors, including the trait to be predicted (e.g., qualitative or quantitative), the quality of the reference data (observed data) set, the spectra quality, the spectra preprocessing, the sample size used to develop the prediction equations, and the validation strategies used for model development and validation (Karoui et al., 2010; Rutten et al., 2010; Ferragina et al., 2015; Bonfatti et al., 2017; Dórea et al., 2018). In addition, directly predicted traits (e.g., milk fat) usually has a significant signal in the spectra data, whereas indirectly predicted traits (e.g., feed efficiency and methane emission) the signal in the spectra is associated to complex traits through compounds found in the scanned products (milk or meat, for example) (Ferragina et al., 2015). The number of research studies implementing alternative methods to predict complex traits in livestock systems is small and, therefore, more investigation using a different analytical approach, sample size, data pretreatment, and variable selection, is important to shed light on the predictive analytics of complex phenotypes.

### Validation Strategies

**FIGURE 2:**
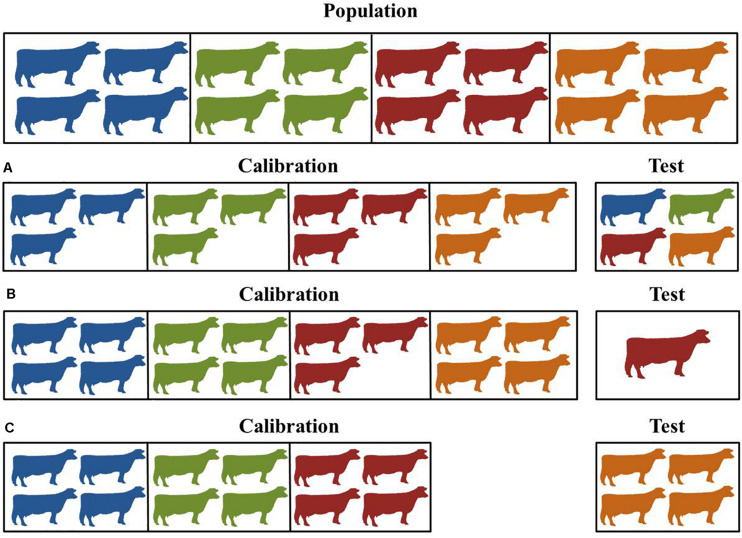
Example of validation strategies employed in the publish papers retrieved from Web of Science. **(A)** split-data or k-fold cross-validation, **(B)** leave-one-out cross-validations, and **(C)** leave-one-group-out cross-validation. Colors represent animals from different herds.

Infrared spectrometry data (MIR and NIR) have been stated as an important source of information to generate many novel complex phenotypes in dairy and beef cattle (Prevolnik et al., 2004; De Marchi et al., 2014; McParland and Berry, 2016; Gengler et al., 2016; Chapman et al., 2019). To predict such phenotypes, robust models or equations must be developed using a training dataset that represents the population variability. The approval that ensures prediction quality of the developed models is given through their performance when implemented in a validation dataset, ideally, an external dataset not previously utilized for model development. However, defining the external validation dataset is a non-trivial task in some cases, since the dependency between training and validation sets needs to be reduced as much as possible. The main reason for reducing such dependence between training and validation sets is the need to approximate prediction quality from model validation to real-life implementation, in which very little dependency between training and validation set will occur. In this review, we considered as an external validation set, all datasets in which some level of dependency between training and validation set was broken. For example, research trials using multiple herds where one herd or trial was removed from the dataset to validate the model. Although this is the desired validation strategy, in some situations, it is difficult to define an external validation set due to the hierarchical structure of the dataset. In such case or when datasets are small, internal validation is usually performed, which was the most used strategy in the papers reviewed here (67 out of 113). In this review, we considered internal validation the studies in which hierarchical structure in the dataset (e.g., country, herd, dietary effect, etc.) was not considered for data split strategies (holdout, leave-one-out, k-fold). Therefore, to create the training and internal validation datasets the reviewed studies used one of the data-splitting techniques: holdout (22 studies), leave-one-out cross-validation (20 studies), and k-fold cross-validation (25 studies). An example of each validation strategy technique used for model validation is depicted in Figure 2.

Briefly, in the holdout strategy (Figure 2A), given that animals are sourced in different herds, about 80% of the dataset is randomly used in the model training and the remaining 20% is used for model validation (Stone, 1974; Picard and Berk, 1990). When holdout is adopted, the test error rate can be highly variable depending on which sample is assigned in the training and validation set (Hastie et al., 2009). Leave-one-out cross-validation is an alternative to the holdout approach and involves splitting the dataset into two parts in which *N – 1* observations are used to train the model and a single sample *N* is used to validate the model (Figure 2B). The splitting is iterated *N* times until all the observations were used in the validation and the model performance is averaged across the *N* validation sets. Thus, the test error in leave-one-out cross-validation is highly variable compared to holdout, because the training dataset contains almost the same number of observations (*N – 1*) as the entire dataset (Hastie et al., 2009). Leave-one-out is commonly used when the sample size is small and there is concern about the limited size of the calibration set (Gianola and Schön, 2016). The idea of leave-one-out cross-validation technique can also be adapted to perform leave-one-herd-out (e.g., group, farm, trial, year, among others), which can be a good strategy to reduce data dependence between training and validation dataset (Figure 2C). The k-fold cross-validation is performed by dividing the entire dataset into k disjoint sets of approximately equal size, usually randomly, in which *k – 1* sets are used in the training and one set is used to validate the model. Such process is repeated *k* times until all *k* sets were used in the validation and the model performance is averaged across all *k* validation sets (Stone, 1974; Hastie et al., 2009). This process can be repeated many times, wherein each iteration different samples are assigned in both training and validation sets. Due to the larger validation dataset assigned in k-fold cross-validation as well as the test error averaged across the *k* different subsets, the test error is less sensitive to the partition of the dataset than in the leave-one-out cross-validation (Hastie et al., 2009). The drawback of the three procedures adopted to split the dataset, except for the leave-one-herd-out strategy, is that animals from the same herd or multiple records from the same animal will be present on the training and validation set, creating dependence between them.

Studies comparing different validation strategies using spectra data confirmed the hypothesis that prediction quality was inflated according to the split-data strategy employed to externally validate the model (Shetty et al., 2017b; Dórea et al., 2018; Lahart et al., 2019; Luke et al., 2019; Smith et al., 2019; Wang and Bovenhuis, 2019). Therefore, evaluating the fitted model using only internal validation is not recommended. The performance of the model fitting will be better than it should be, resulting in greater model precision and accuracy than if a true external validation is used, but the prediction accuracies in the external validation set are more realistic and quite often observed in practice. Several authors (32 out of the 112 papers reviewed here) reported that an independent dataset was used to externally validate the model’s performance. However, by reviewing the papers from the 32 studies only seventeen fully performed external model validation. In those studies, the authors validated the models’ performance using an external/independent dataset, which an entire farm, herd, trial, year, study, region, dataset, or batch was removed from the training dataset. The remaining studies (15) only assigned records by animal, individual records, or lactations either randomly or not from the entire dataset to external validate the model. Such strategy does not produce a good independent dataset to test the model against real-life implementation because animals from the same herd or group can be present on the training and validation set. Fourteen out of 112 papers did not report if model performance was evaluated, only stated that cross-validation was employed, or the procedure was not clearly described. Therefore, it is a good practice to use external validation (if large sample size is available), based on data from a different farm, herd, trial, year, region, or batch, before full deployment of such predictive models.

## Concluding Remarks

Important advances have been made in the nutrition, reproduction, management, and molecular breeding techniques of beef and dairy cattle in recent years. However, efficient and precise phenotyping remains a bottleneck and, therefore, modern high-throughput techniques should be developed, improved, and applied to take full advantage of the advancements performed in the different animal knowledge fields. Of the techniques currently available, this review summarized the applications of MIR and NIR spectrometry as a novel high-throughput phenotyping technique to generate complex phenotypes in dairy and beef cattle. Furthermore, it presented an overview, status update, and insights into the use of such techniques and the data mining strategies employed to predict the phenotypes of interest. The majority of studies compiled have demonstrated the capability and power of MIR and NIR technique to generate complex traits such as feed efficiency, methane emissions, energy balance, health, and meat quality from different biological samples routinely accessed, without additional cost and at the animal-level. Therefore, these phenotypes would be widely explored in dairy and beef cattle for on-farm decision-making, management, and breeding purpose. MIR and NIR spectrometry has important advantages compared to gold standard methods such as speed, low cost, non-invasive, non-destructive, and potential for in-line application; however, for the implementation of these high-throughput techniques into livestock operations, numerous issues regarding the modeling methodology must be considered. Few studies have used a large dataset as well as machine learning or Bayesian techniques to develop the calibrations models. Therefore, larger datasets and modern data mining approaches should be investigated to improve predictive ability and to confirm the existing calibration models.

## Author Contributions

TB and JD equally contributed direct and intellectual to the work and approved it for publication. Both authors contributed to the article and approved the submitted version.

## Conflict of Interest

The authors declare that the research was conducted in the absence of any commercial or financial relationships that could be construed as a potential conflict of interest.
