# Erratum: Infrared Spectrometry as a High-Throughput Phenotyping Technology to Predict Complex Traits in Livestock Systems

**DOI:** 10.3389/fgene.2020.628196

**Published:** 2020-12-07

**Authors:** Frontiers Production Office

**Affiliations:** Frontiers Media SA, Lausanne, Switzerland

**Keywords:** beef cattle, dairy cattle, near-infrared, novel phenotypes, mid-infrared, spectral information

Due to a production error, an incorrect reference citation “Vanlierde et al. (2016)” was inserted in Table 5. It should be “Visentin et al. (2016).” The corrected Table 5 appears below.

**Table 5:** Number of samples (N) and coefficient of determination in the validation set for mineral contents using partial least square methodology.

**References**	**N**	**Breed**	Validation^*^	**Ca**	**K**	**Mg**	**Na**	**P**
Soyeurt et al. (2009)	92	Multibreed	LOOCV	0.87	0.36	0.65	0.65	0.85
Gottardo et al. (2015)	208	–	10-F CV^b^	0.55	–	–	–	–
Toffanin et al. (2015)	208	Holstein	LOOCV	0.53^c^	–	–	–	0.70^c^
Bonfatti et al. (2016)	689	Simental	10-F CV	0.48	0.41	0.46	–	0.43
Visentin et al. (2016)	923	Multibreed	R-Tr/Te^b^	0.67	0.69	0.65	0.40	0.68
Malacarne et al. (2018)^a^	153	Holstein	Tr/Te^b^	0.25	0.34	0.26	0.25	0.53
Franzoi et al. (2019)^b^	93	Holstein	CV	0.79	0.55	0.68	0.75	0.87
Fleming et al. (2019)	986	Multibreed	10- FCV	0.25	–	–	–	–

a *Bulk milk samples*.

b *Backward interval partial least squares (BiPLS), number of folds (n-F) in the cross-validation, leave-one-out cross-validation (LOOCV), train and test cross-validation defined by splitting the data set randomly (R-Tr/Te), external or independent validation*.

c *Correlation coefficient (r) transformed to coefficient of determination (R2)*.

* *The validation strategy defined as “CV” was assigned for the reviewed paper that did not completely describe the validation method adopted or the authors defined that cross-validation was employed*.

The publisher apologizes for this mistake. The original article has been updated.
